# Implementation of a Care Management Program for Low-Income Older Adults

**DOI:** 10.1093/geroni/igaa057.2918

**Published:** 2020-12-16

**Authors:** Anna Schloen, Helen Grimaldi

**Affiliations:** CJE SeniorLife, Chicago, Illinois, United States

## Abstract

The focus of this session will be on the implementation of a new care management program in Chicago for low income older adults. Framed by the Exploration, Installation, Initial Implementation, and Full Implementation framework, the presenter will first discuss the process and outcomes of a needs assessment which informed the program. The presenter will share strategies and lessons learned from getting the program off the ground and initial lessons learned which informed the full program as it is operated today. In 2 years, the program has grown from 1 FTE to 4.5 FTE and to 250 clients. The presenter will provide strategies for managing growth while maintaining quality care. Finally, the presenter will provide information on the program's collaboration with a researcher to enhance evidence-based service delivery within the care management program. Participants will learn specific strategies they can take back to their own communities to implement care management programs.

